# Mid-term Psychiatric Outcomes of Patients Recovered From COVID-19 From an Italian Cohort of Hospitalized Patients

**DOI:** 10.3389/fpsyt.2021.667385

**Published:** 2021-06-10

**Authors:** Carla Gramaglia, Eleonora Gambaro, Mattia Bellan, Piero Emilio Balbo, Alessio Baricich, Pier Paolo Sainaghi, Mario Pirisi, Giulia Baldon, Sofia Battistini, Valeria Binda, Alessandro Feggi, Martina Gai, Eleonora Gattoni, Amalia Jona, Luca Lorenzini, Debora Marangon, Maria Martelli, Pierluigi Prosperini, Patrizia Zeppegno, The NO-MORE COVID Group, Gian Carlo Avanzi

**Affiliations:** ^1^Dipartimento di Medicina Traslazionale, Università del Piemonte Orientale UPO, Novara, Italy; ^2^Azienda Ospedaliero Universitaria Maggiore della Carità, Novara, Italy

**Keywords:** COVID-19, anxiety, depression, patients, follow-up, mid-term sequelae

## Abstract

**Background:** Although the usual primary clinical manifestation of Coronavirus disease (COVID-19) is respiratory, several non-respiratory symptoms have been described, including neuropsychiatric ones. The aim of this study was to investigate the mid-term mental health outcomes in patients recovered from COVID-19, 3–4 months after discharge from the University Hospital Maggiore della Carità, Novara, Italy. Furthermore, we investigated the possible association of the mid-term mental health consequences of the COVID-19 infection with patients' clinical current status, persistent physical impairment and severity of acute phase of the disease.

**Methods:** Prospective study involving 238 individuals recovered from COVID-19. In the context of a multi-disciplinary approach, patients' assessment included both a clinical interview performed by an experienced psychiatrist, trained in the use of the Mini-International Neuropsychiatric Interview to assess the presence of anxiety and depressive symptoms and self-administered questionnaires: Beck Anxiety Inventory (BAI), Beck Depression Inventory-II (BDI-II), Resilience Scale for Adults (RSA), Impact of Event Scale (IES).

**Results:** At the psychiatric assessment 32.9 and 29.5% of participants showed anxiety and depressive symptoms, respectively. Changes in appetite and sleep patterns emerged for 15.6 and 31.2% of patients. According to the self-administered questionnaires, 7.1% of participants had moderate-severe anxiety levels (BAI), while 10.5% had mild to severe depression (BDI-II). Twenty-six (11%) participants were referred to further psychiatric consultation. Psychiatric symptoms showed no correlation with acute COVID-19 severity; in our sample patients with depressive symptoms at the clinical interview, as well as those with mild to severe levels of depression according to BDI-II scores, had lower forced expiratory volume in the 1st second (FEV1) values than those without and greater odds for persistent, poor tolerance for physical efforts.

**Conclusions:** As could be expected, an approach including both a psychiatric interview and the use of self-administered questionnaires is likely to capture the psychiatric outcome of patients recovered from COVID-19 better than questionnaires alone. Anxiety and depressive symptoms at follow-up had no correlation with the severity of COVID acute manifestations, but rather with ongoing and persistent physical symptoms. Further studies and longer follow-up duration will allow a better understanding of the complex relationship between residual physical symptoms, quality of life and psychological health.

## Introduction

Several unpredictable and varied non-respiratory symptoms, including neuropsychiatric ones, have been described for severe acute respiratory syndrome coronavirus 2 (SARS-CoV-2), even though the usual and primary clinical manifestation of Coronavirus disease (COVID-19) is respiratory (1–7). Besides strictly neurological symptoms, and persistent consequences on cognitive function (8), psychopathological symptoms might appear as well in patients with COVID-19 either acutely ill or recovered. Hypotheses concerning the etiopathogenesis of neuropsychiatric presentations in individuals with COVID-19 include both direct (central nervous system tropism) and indirect mechanisms (i.e., respiratory failure and related medical procedures such as intubation, cytokine storm, host immune response, viral infection-induced cerebrovascular accidents), whose contribution has yet to be established and which are hard to disentangle (9). Furthermore, adverse mental health outcomes may be related to the overall pandemic-associated psychological distress (fear or experience of illness, uncertainty, stigma, social strain) (10, 11), which has been reported to be highest in patients with COVID-19 compared to the general public and healthcare workers, and even more likely in the vulnerable group of psychiatric patients (12).

As shown by previous outbreaks of coronaviruses such as those responsible of Middle East respiratory syndrome coronavirus infection (MERS) or severe acute respiratory syndrome (SARS) (13, 14), mental health-related symptoms (depression, anxiety, reduced quality of life, Post Traumatic Stress Disorder, etc.), as well as persistent impairment in pulmonary function, muscle weakness, fatigue and pain, can be relevant issues for patients, also in the mid-and long-term. Therefore, it is clear that a timely assessment of psychiatric symptoms in individuals with COVID-19 is necessary for a better prognostic outcome (5). To achieve this objective, attention needs to move from acute care settings to the post-acute care of patients recovered from COVID-19. While neuro-psychiatric symptoms acutely arising during the infection (such as delirium, transient psychotic disorders, acute stress, sleep disorders, anxiety) have received more attention (15, 16), the mid- and long-term consequences in patients recovered from COVID-19 are still unknown and poorly investigated (17).

A recent review of psychiatric symptoms or morbidities associated with COVID-19 in infected patients and non-infected groups (psychiatric patients, healthcare workers and non-healthcare workers) found that only two studies, out of the 43 included, evaluated patients with confirmed COVID-19 infection. Two studies found a high level of post-traumatic stress symptoms (PTSS) (96.2%) and significantly high prevalence of depressive symptoms (29.2%) in newly recovered patients (18, 19). Rates of Post-Traumatic Stress Disorder (especially intrusive images) have been found to be particularly high among patients with more severe COVID-19 related symptoms (20). Different studies have reported rates of sleep disturbances/disorders or insomnia ranging from 17.7 to 35.1% (21–25). The prevalence of depressive and anxiety symptoms among patients recovered from COVID-19 was investigated in patients living in different countries and rates widely varied as well, from 4.3 to 37.3% for depression and from 6.5 to 47.8% for anxiety (21–27).

Interestingly, perceived discrimination, stigma, and social support were suggested as predictors of mental health outcomes, both in patients recovered and in hospital inpatients with confirmed COVID-19 (25, 28).

From the available literature, it emerges that the understanding of the mid- and long-term impact of COVID-19 is far from being complete, not only in the context of a multidisciplinary approach, but even more when focusing on mental health/psychiatric outcomes (29). Therefore, it is of crucial importance to deepen the neuropsychiatric aspects of patients recovered from COVID-19, possibly including the assessment of psychosocial impact as well as neuroinflammation. As described above, mid-term psychiatric outcomes of individuals recovered from COVID-19 are still poorly investigated (21–27), although an early intervention for the prevention of these sequelae would be recommendable, as it is, for instance, for the psychological consequences of the pandemic at the general population level (30). Moreover, regarding the available literature, it should be noted that research articles about this topic are highly heterogeneous as far as sample size, inclusion and exclusion criteria and follow-up duration (only a few studies followed-up patients for more than 2 months) are concerned; patients' assessment is based mainly on different kind of assessment tools and self-administered questionnaires which do not equate a clinical diagnosis (22, 23, 26, 27). The importance of the topic is further underscored by the fact that recent literature introduced the term “Long COVID” in order to describe people recovered from COVID-19, but still having lasting symptoms, such as fatigue, muscle weakness, sleep difficulties, anxiety or depression (21, 31). It has been suggested that scientific studies must better identify the heterogeneity of Long COVID, considering that it might represent another public health crisis (32).

In the context of a multi-disciplinary follow-up project (33), our aim was to investigate the mid-term psychiatric outcomes (anxiety, depressive symptoms, post-traumatic stress symptoms, resilience) of patients recovered from COVID-19, followed-up 3 to 4 months after discharge from hospital. For this purpose, our assessment included both a thorough clinical interview performed by an experienced psychiatrist and the use of self-administered, validated questionnaires. Previously published data from this project have already highlighted that 17% of patients undergoing the multi-disciplinary assessment showed post-traumatic stress symptoms (moderate-severe scores at the Impact of Event Scale); in the current paper, we will focus on anxiety and depressive symptoms.

Considering the currently available research about this topic, we were particularly interested in assessing the possible differences between anxiety and depressive symptoms as identified by the clinical interview performed by an experienced psychiatrist and as assessed with self-administered screening questionnaires. Furthermore, we investigated the possible association of the mid-term mental health consequences of the COVID-19 infection with patients' clinical current status, persistent physical impairment and severity of acute phase of the disease. We postulated that the persistence of physical symptoms, especially respiratory ones, could be the main driver in the maintenance of the mid-term mental health consequences of the COVID-19 infection, rather than the severity of the acute disease.

## Methods

A prospective study was performed; more details about the methodology of the study and recruitment procedures can be found elsewhere (33). We contacted by phone, around 4 months after discharge [median time after discharge 131 (119–145) days], 767 consecutive patients, older than 18 years of age, admitted for COVID-19 and then discharged from the University Hospital “AOU Maggiore della Carità” in Novara (Northern Italy) between 1^st^ March 2020 and 29^th^ June 2020. Out of these 767 patients, 35 (4.6%) had died since hospital discharge and 494 (64.4%) declined participation. We eventually enrolled 238 (31.0%) subjects who gave their informed consent to participate. No exclusion criterion was applied, except for unwillingness to provide consent (further details about enrollment have been described elsewhere) (33). An electronic case report form was generated using the Research Electronic Data Capture software (REDCap, Vanderbilt University) for clinical data collection, and a unique pseudonymized code was attributed to each patient included into the study. The study protocol was approved by the local ethical committee (Comitato Etico Interaziendale Novara; IRB code CE 117/20) and research was conducted according to the principles of the Declaration of Helsinki.

Information was gathered about: socio-demographic features, symptoms during the acute COVID-19 phase, comorbidities, home medications and complications during the hospital stay. The multi-disciplinary team involved in this clinical and research project performed a thorough assessment of patients who, a few days after the phone contact, were invited to attend an outpatient visit at the hospital, including the following: internal medicine visit to ascertain patients' symptoms at follow-up and new diagnoses received after hospital discharge; pneumology visit including spirometry; physiatric visit with physical performance tests; psychiatric assessment, including a thorough psychiatric interview and self-administered questionnaires which were filled in during the hospital stay for the outpatient visit, as detailed below.

In this paper, we specifically focused on the following socio-demographic and clinical variables: age, gender, smoking attitude, history of chronic obstructive pulmonary disease (COPD), hypertension, diabetes, ischemic cardiac disease, obesity, type of oxygen support during hospitalization, Intensive Care Unit (ICU) admission. At follow-up we recorded: the forced expiratory volume in the 1^st^ second (FEV1), the forced vital capacity (FVC), the diffusing capacity of the lung for carbon monoxide (DLCO), the complaint of dyspnea and the perceived tolerance to physical efforts.

Regarding psychiatric assessment at follow-up, patients were interviewed face-to-face by an experienced psychiatrist, trained in the use of the Mini-International Neuropsychiatric Interview (MINI) (34), with structured and unstructured questions about current mental health status; previous psychiatric history (being already treated by psychiatric services; a history of anxiety and/or depression); depressive and anxiety symptoms, changes in sleep and eating patterns since discharge from hospital. Interviewers rated the questions concerning depressive and anxiety symptoms, and changes in sleep and eating patterns as follows: a “yes” answer was attributed in those cases where a clinically relevant symptom emerged, a “no” answer in all other cases. Information was recorded about the outcome of the psychiatric consultation (no further indication; referral to further psychiatric care for support or medication). Furthermore, patients were asked to fill in the following self-administered measures during their hospital stay for the outpatient, multidisciplinary follow-up visit: Beck Anxiety Inventory (BAI), Beck Depression Inventory (BDI-II), Resilience Scale for Adults (RSA) and the Impact of Event Scale (IES). The validated Italian version was used for all the self-administered measures.

### The Mini-International Neuropsychiatric Interview (MINI)

The MINI (34) is a brief structured interview developed on the basis of DSM-IV and ICD-10 in order to identify psychiatric diagnosis. The administration time is approximately 15 min and it was designed with the aim to be a short, simple, but highly sensitive tool. For our study, we adopted the A, E, O modules to screen for major depressive disorder, panic disorder, and generalized anxiety disorder.

### Beck Anxiety Inventory (BAI)

It is a self-report 21-item questionnaire measuring the emotional, physiological and cognitive symptoms of anxiety as well as its severity (35). Each item is rated on a 4-points scale (from 0 to 3). The following levels of anxiety are identified according to the total score: minimum (0–21); moderate (22–35); high (>36). For the subsequent analyses, we grouped together participants with a total score higher that 21; in other words, we categorized participants in those with minimum BAI scores and those with higher scores (moderate-severe). BAI's internal consistency measured by Cronbach Alpha is 0.94 (36).

### Beck Depression Inventory (BDI-II-II)

It is one of the most used measures for depression (37). It is a self-report questionnaire with 21-item rated on a 4-points scale (from 0 to 3), asking about symptoms in the last 2 weeks. Total scores suggest different levels of depression: minimum (0–13); mild (14–19); moderate (20–28); severe (29–63). Internal consistency measured by Cronbach Alpha is 0.86 for the mental component, 0.65 for the somatic component (38). For the subsequent analyses, we grouped together participants with a total score higher than 13; in other words, we categorized participants in those with minimum BDI-II scores and those with higher scores (mild-moderate-severe).

### Resilience Scale for Adults (RSA)

It is a self-administered scale consisting of 33 items that examine intra- and inter-personal protective factors deemed capable of facilitating adaptation in the face of psychosocial adversity (39). The factor analysis identified six factors: positive perception of self, positive perception of the future, social competence, structured style, family cohesion and social resources. There are no cut-offs for this scale; the higher the total score, the greater is the person's overall resilience. Internal consistency evaluated by Cronbach alpha is 0.86 (40).

### Impact of Event Scale (IES)

It is a 15-item self-rated 4-point scale based on how often an event has occurred in the past 7 days (0 indicates not at all; 1, rarely; 3, sometimes; 5, often), in order to assess the presence of post-traumatic stress (PTS) symptoms (41). Besides the IES total subjective stress score, 2 subscales are identified. One subscale (7 items) measures intrusive symptoms (intrusive thoughts, nightmares, intrusive feelings and imagery), with scores ranging from 0 to 35; the other subscale (8 items) measures avoidance symptoms (numbing of responsiveness and avoidance of feelings, situations, or ideas), with scores ranging from 0 to 40. Internal consistency coefficients for intrusion is 0.84, for avoidance is 0.71 (42).

### Statistical Analysis

Enrolled patients were 238, but full data for the psychometric questionnaires were available for 237 of them (1 patients did not fill all of them and hence was excluded from the current analyses).

Data were analyzed using the statistical software MedCalc v19.2.6 (43). Normality was assessed by Shapiro-Wilk test. The measures of centrality and dispersion chosen for continuous variables were medians and [25^th^-75^th^ percentile].

We firstly established the prevalence of anxiety and depressive symptoms according to the clinical interview and we assessed the level of anxiety and depression as measured by the BAI and BDI-II. We then evaluated the possible associations between persistence of anxiety and depressive symptoms (both as assessed at the clinical interview and according to BAI and BDI-II) and the following continuous or categorical variables, related to: patients' demographic features (age and gender), length of hospital in-stay, patients' comorbidities such as COPD, hypertension, diabetes, ischemic cardiac disease, obesity, previous history of anxiety and/or depression, smoking attitude, being already treated by psychiatric services, questionnaire scores (IES and RSA); severity of acute phase (ICU admission and modality of oxygen delivery during hospitalization); persistent physical sequelae (DLCO, FEV1, FVC, presence of dyspnea, poor tolerance to physical efforts, changes in appetite and sleep patterns); being recommended further psychiatric consultation. For this purpose, we run a univariate analysis, first: comparisons between groups were performed by the Mann-Whitney test for continuous variables (age, DLCO, FEV1, FVC, RSA total score). Categorical variables were reported as frequencies and percentages and analyzed through the Pearson's chi-square or Fisher's exact test, as appropriate. All the associations with a *p* value <0.20 were then included in logistic regression models.

## Results

### General Features and Outcome of the Psychiatric Interview

Patients' general features have already been described elsewhere (33). According to the information gathered in the context of the psychiatric interview, 32 participants (13.5%) were already treated by psychiatric services; 11 (4.6%) had a previous history of anxiety and/or depression.

Changes in appetite and sleep patterns emerged at the clinical interview for 37 (15.6%) and 74 (31.2%) of patients, respectively.

Data about anxiety and depressive symptoms at the psychiatric interview are described in the sections below. According to BAI and BDI-II scores, patients were categorized in two groups, as described in the methods; 17 (7.1%) participants had moderate-severe anxiety levels according to the BAI, while the BDI-II found mild to severe depression scores in 25 (10.5%) patients.

After the psychiatric interview and assessment, 26 (11%) participants were referred to further psychiatric consultation.

### Anxiety at the Psychiatric Interview

Anxiety symptoms were found in 78 patients (32.9%) at the clinical interview. When comparing patients with and without anxiety symptoms at the psychiatric interview, no difference was found in BAI categorization (*p* = 0.522). On the other hand, differences were found in depressive symptoms, sleep and appetite patterns as assessed at the psychiatric interview, being already treated by psychiatric services, being recommended further psychiatric consultation (Table 1). Moreover, the univariate analysis found that participants with persistent anxiety symptoms at the psychiatric interview were significantly younger than those without (median age of those with anxiety vs. those without, 56 vs. 62, *p* = 0.02) (Table 2); nonetheless, this result was not supported by the logistic regression analysis (Table 3).

**Table 1:** Anxiety as assessed at the psychiatric interview and with the BAI: chi squared test.

		**Anxiety at the clinical interview**		***p***	**BAI total score**		***p***
		**Yes**	**No**		**Minimum (<22)**	**Moderate-high (≥22)**	
		**N (%)**	**N (%)**		**N (%)**	**N (%)**	
IES total score	Minimal (0–8)	45 (57.70)	90 (56.60)	0.641	133 (60.45)	2 (11.76)	** <0.001**
	Low (9–25)	19 (24.36)	42 (26.41)		56 (25.45)	5 (29.41)	
	Moderate (26–43)	11 (14.10)	16 (10.07)		23 (10.45)	4 (23.53)	
	High (>44)	3 (3.84)	11 (6.92)		8 (3.64)	6 (35.29)	
Depression at the clinical interview	Yes	46 (58.97)	24 (15.09)	** <0.001**	65 (29.55)	5 (29.41)	1.000
	No	32 (41.03)	135 (84.49)		155 (70.45)	12 (70.59)	
Sent for further psychiatric referral/consultation	Yes	24 (30.77)	2 (1.28)	** <0.001**	24 (10.90)	2 (11.76)	1.000
	No	54 (69.23)	157 (98.74)		196 (89.10)	15 (88.24)	
History of anxiety and/or depression	Yes	6 (7.70)	5 (3.14)	0.185	10 (4.55)	16 (94.11)	0.801
	No	72 (92.30)	154 (96.86)		210 (94.45)	1 (5.88)	
Already treated by psychiatric services	Yes	27 (34.61)	5 (3.14)	** <0.001**	30 (13.63)	2 (11.76)	1.000
	No	51 (65.38)	154 (96.85)		190 (86.64)	15 (88.24)	
Exercise intolerance/poor tolerance to physical efforts	Yes	57 (73.07)	130 (81.76)	0.131	44 (0.20)	6 (35.40)	0.212
	No	21 (26.93)	29 (18.24)		176 (0.80)	11 (64.70)	
Gender	Male	40 (51.28)	101 (63.52)	0.091	131 (59.55)	10 (58.82)	1.000
	Female	38 (48.72)	58 (36.48)		89 (40.45)	7 (41.18)	
Sleep problems at the clinical interview	Yes	46 (58.97)	28 (17.61)	** <0.001**	68 (30.90)	6 (35.40)	0.787
	No	32 (41.03)	131(82.39)		152 (69.10)	11 (64.70)	
Appetite problems at the clinical interview	Yes	24 (30.77)	13 (8.18)	** <0.001**	34 (15.5)	3 (17.6)	0.734
	No	54 (69.23)	146 (91.82)		186 (84.5)	14 (82.4)	
COPD	Yes	6 (7.70)	8 (5.03)	0.40	14 (6.36)	0 (0.00)	0.61
	No	72 (92.30)	151 (94.97)		206 (93.64)	17 (100)	
Ischemic heart disease	Yes	3 (3.85)	18 (11.32)	0.09	21 (9.54)	0 (0.00)	0.37
	No	75 (96.15)	141 (88.68)		199 (90.46)	17 (100.00)	
Dyspnea	Yes	5 (6.41)	8 (5.03)	0.76	12 (5.45)	1 (5.88)	1.00
	No	73 (93.59)	151 (94.97)		208 (94.55)	16 (94.12)	
Diabetes	Yes	10 (12.82)	26 (16.35)	0.57	34 (15.45)	2 (11.76)	1.00
	No	68 (87.18)	133 (83.65)		186 (84.55)	15 (88.24)	
Hypertension	Yes	30 (38.46)	67 (42.14)	0.67	93 (42.27)	4 (23.53)	0.20
	No	48 (61.54)	92 (57.86)		127 (57.73)	13 (76.47)	
Obesity	Yes	11 (14.10)	14 (8.80)	0.26	23 (10.45)	2 (11.76)	0.70
	No	67 (85.90)	145 (91.20)		197 (89.55)	15 (88.24)	
Type of oxygen support	NC	29 (37.18)	37 (23.27)	0.15	61 (27.73)	5 (29.41)	0.68
	VM	28 (35.90)	72 (45.28)		95 (43.18)	5 (29.41)	
	CPAP/NIV	14 (17.95)	36 (22.64)		45 (20.45)	5 (29.41)	
	EI	7 (8.97)	14 (8.81)		19 (8.64)	2 (11.76)	
ICU admission	Yes	9 (11.54)	19 (11.95)	1.00	24 (10.91)	4 (23.53)	0.12
	No	69 (88.46)	140 (88.05)		196 (89.09)	13 (76.47)	
Smoking	Never	46 (58.97)	92 (57.86)	0.99	129 (58.64)	9 (52.94)	0.61
	Current	8 (10.26)	17 (10.69)		22 (10.00)	3 (17.65)	
	Former	24 (30.77)	50 (31.45)		69 (31.36)	5 (29.41)	

*BAI, Beck Anxiety Inventory; COPD, Chronic Obstructive Pulmonary Disease; CPAP/NIV, Continuous Positive Airway Pressure/Non-Invasive Ventilation; EI, endo-tracheal intubation; IES, Impact of Event Scale; NC, nasal cannulae; VM, Venturi Mask.*

*The P-values of significant findings are written in bold*.

**Table 2:** Anxiety as assessed at the psychiatric interview and with the BAI: Mann-Whitney test results.

	**Anxiety at the clinical interview**		***p***	**BAI total score**		***p***
	***Yes***	***No***		**Minimum (<22)**	**Moderate-high (≥22)**	
	**Median** **[25th-75th percentile]**	**Median** **[25th-75th percentile]**		**Median** **[25th-75th percentile]**	**Median** **[25th-75th percentile]**	
Age (years)	56.00 [47.00–68.00]	62.00 [52.00–71.00]	**0.02**	61.00 [51.00–71.00]	53.00 [43.25–67.50]	0.18
DLCO (% predicted value)	80.50 [67.00–90.00]	78.00 [69.00–87.00]	0.34	79.00 [69.00–89.00]	74.50 [60.50–87.50]	0.38
FEV1 (% predicted value)	100.00 [89.25–113.75]	101.00 [92.50–111.00]	0.84	101.00 [92.00–112.00]	99.00 [85.50–113.50]	0.70
FVC (% predicted value)	100.00 [90.00–111.00]	97.00 [90.00–108.00]	0.41	98.00 [90.00–109.00]	98.50 [87.00–110.00]	0.97
RSA total score	134.00 [119.25–147.00]	137.00 [118.00–146.00]	0.95	135.00 [120.00–147.50]	134.00 [111.25–143.75]	0.29
Length of hospital in-stay (days)	8 [4–16]	9 [8–11]	0.33	9 [5–16]	7 [4–14]	0.49

*BAI, Beck Anxiety Inventory; DLCO, diffusing capacity of the lung for carbon monoxide; FEV1, forced expiratory volume in the 1st second; FVC, forced vital capacity; RSA, Resilience Scale for Adults.*

*The P-values of significant findings are written in bold*.

**Table 3:** Logistic regression model for anxiety as assessed at the psychiatric interview and with the BAI.

	**Anxiety at the clinical interview**			**BAI Total score (≥22)**	
	**OR (95% CI)**	***p*-value**		**OR (95% CI)**	***p*-value**
Age	0.98 (0.96–1.01)	0.15	Age	0.98 (0.95–1.03)	0.53
Exercise intolerance/Poor tolerance to physical efforts	1.66 (0.85–3.24)	0.14	Exercise intolerance/Poor tolerance to physical efforts	1.25 (0.36–4.37)	0.72
Gender	0.68 (0.38–1.22)	0.19	ICU admission	1.45 (0.34–6.02)	0.61
History of anxiety and depression	2.57 (0.71–9.29)	0.15	IES Total score category	3.14 (1.88–5.25)	** <0.001**
Oxygen support	0.85 (0.62–1.66)	0.31	Hypertension	0.52 (0.15–1.83)	0.31
Ischemic heart disease	0.43 (0.12–1.58)	0.20			

*BAI, Beck Anxiety Inventory; ICU, Intensive Care Unit; IES, Impact of Event Scale.*

*The P-values of significant findings are written in bold*.

### Anxiety According to BAI Score

As described in the methods, patients were categorized in two groups according to BAI scores (minimal vs. higher scores). The two groups showed no statistically significant difference in changes in sleep and appetite patterns, being already treated by psychiatric services, being sent for further psychiatric consultation (Table 1). The two groups differed in post-traumatic stress symptoms, as patients in the moderate-severe anxiety BAI category were more likely to fall in the moderate-high IES score category (Table 1). Furthermore, no statistically significant differences were found between the two groups regarding any of the socio-demographic and clinical continuous variables we gathered (age, DLCO, FEV1, FVC, IES score, RSA Total score, see Table 2).

The logistic regression yielded no statistically significant result except for the IES score (*P* = 0.0001; OR 3.14, 95% CI 1.88–5.25) (Table 3).

### Depressive Symptoms at the Psychiatric Interview

Depressive symptoms at the clinical interview were found in 70 patients (29.5%). Patients with depressive symptoms according to the psychiatric interview, compared to those without, more frequently had mild to severe BDI-II scores. Statistically significant differences between the two groups included anxiety and changes in sleep and appetite patterns as assessed at the psychiatric interview; being suggested further psychiatric consultation. Further associations emerged with female gender (*p* = 0.0001) and COPD (*p* = 0.03) (Table 4).

**Table 4:** Depressive symptoms as assessed at the psychiatric interview and with the BDI-II: chi squared test.

		**Depressive symptoms at the clinical interview**		***p***	**BDI-II total score**		***p***
		**Yes**	**No**		**Minimum ≤13**	**Mild to severe >13**	
		**N (%)**	**N (%)**		**N (%)**	**N (%)**	
IES total score	Minimal (0–8)	39 (55.72)	96 (57.48)	0.97	121 (57.07)	14 (56)	0.53
	Low (9–25)	18 (25.72)	43 (25.75)		54 (25.47)	7 (28)	
	Moderate (26–43)	9 (12.85)	18 (10.78)		23 (10.85)	4 (16)	
	High (>44)	4 (5.71)	10 (5.99)		14 (6.61)	0 (0)	
Sent for further psychiatric referral/consultation	Yes	21 (30.)	5 (2.99)	** <0.001**	17 (8.02)	9 (36)	** <0.001**
	No	49 (70)	162 (97.01)		195 (91.98)	16 (64)	
History of anxiety and/or depression	Yes	64 (91.42)	162 (9.7)	0.0876	6 (2.83)	5 (20)	**0.001**
	No	6 (8.58)	5 (90.3)		206 (97.17)	20 (80)	
Already treated by psychiatric services	Yes	22 (31.42)	10 (5.99)	** <0.001**	24 (11.32)	8 (32)	**0.009**
	No	48 (68.58)	157 (94.01)		188 (88.68)	17 (68)	
Exercise intolerance/poor tolerance to physical efforts	Yes	19 (27.14)	31 (18.56.)	0.16	40 (18.87)	10 (40)	**0.02**
	No	51 (72.86)	136 (81.44)		172 (81.13)	15 (60)	
Gender	Male	28 (40)	113 (67.66)	** <0.001**	132 (62.26)	9 (36)	**0.017**
	Female	42 (60)	54 (32.34)		80 (37.74)	16 (64)	
Sleep problems at the clinical interview	Yes	39 (55.71)	35 (20.96)	** <0.001**	59 (27.83)	15 (60)	**0.002**
	No	31 (44.29)	132 (79.04)		153 (72.17)	10 (40)	
Appetite problems at the clinical interview	Yes	23 (32.86)	14 (8.38)	** <0.001**	29 (13.68)	8 (32)	**0.035**
	No	47 (67.14)	153 (91.62)		183 (86.32)	17 (68)	
Anxiety at the clinical interview	Yes	46 (65.71)	32 (19.16)	** <0.001**	62 (29.24)	16 (64)	**0.0049**
	No	24 (34.29)	135 (80.84)		150 (70.76)	9 (36)	
COPD	Yes	8 (11.43)	6 (3.59)	**0.03**	12 (5.66)	2 (8.00)	0.65
	No	62 (88.57)	161 (96.41)		200 (94.34)	23 (92.00)	
Ischemic heart disease	Yes	4 (5.71)	17 (10.18)	0.32	20 (9.43)	1 (4.00)	0.71
	No	66 (94.29)	150 (89.82)		192 (90.57)	24 (96.00)	
Dyspnea	Yes	6 (8.57)	7 (4.19)	0.21	12 (5.66)	1 (4.00)	1.00
	No	64 (91.43)	160 (95.81)		200 (94.34)	24 (96.00)	
Diabetes	Yes	8 (11.43)	28 (16.77)	0.33	35 (16.51)	1 (4.00)	0.14
	No	62 (88.57)	139 (83.23)		177 (83.49)	24 (96.00)	
Hypertension	Yes	23 (32.86)	74 (44.31)	0.11	84 (39.62)	13 (52.00)	0.28
	No	47 (67.14)	93 (55.69)		128 (60.38)	12 (48.00)	
Obesity	Yes	7 (10.00)	18 (10.78)	1.00	20 (9.43)	5 (20.00)	0.16
	No	63 (90.00)	149 (89.22)		192 (90.57)	20 (80.00)	
Type of oxygen support	NC	27 (38.57)	39 (23.35)	0.10	57 (26.89)	9 (36.00)	0.61
	VM	27 (38.57)	73 (43.72)		89 (41.98)	11 (44.00)	
	CPAP/NIV	12 (17.15)	38 (22.75)		47 (22.17)	3 (12.00)	
	EI	4 (5.71)	17 (10.18)		19 (8.96)	2 (8.00)	
ICU admission	Yes	7 (10.00)	21 (12.57)	0.66	24 (11.32)	4 (16.00)	0.51
	No	63 (90.00)	146 (87.43)		188 (88.68)	21 (84.00)	
Smoking	Never	43 (61.43)	95 (56.89)	0.53	122 (57.55)	16 (64.00)	0.52
	Current	5 (7.14)	20 (11.98)		24 (11.32)	1 (4.00)	
	Former	22 (31.43)	52 (31.13)		66 (31.13)	8 (32.00)	

*BDI-II, Beck Depression Anxiety Inventory-II; COPD, Chronic Obstructive Pulmonary Disease; CPAP/NIV, Continuous Positive Airway Pressure/Non-Invasive Ventilation; EI, endo-tracheal intubation; IES, Impact of Event Scale; NC, nasal cannulae; VM, Venturi Mask.*

*The P-values of significant findings are written in bold*.

Participants with depressive symptoms, as identified by the clinical interview, had lower FEV1 values than those who did not (*p* = 0.0231; median 97 [90.0–104.7] vs. 102 [93.5–113.5]) (Table 5), but showed no difference in the other continuous variables assessed (age, DLCO, FVC, RSA total score).

**Table 5:** Depressive symptoms as assessed at the psychiatric interview and with the BDI-II: Mann-Whitney test results.

	**Depressive symptoms at the clinical interview**		***p***	**BDI-II total score**		***p***
	***Yes***	***No***		**Minimum (≤13)**	**Medium-severe (>13)**	
	**Median** **[25th–75th percentile]**	**Median** **[25th–75th percentile]**		**Median** **[25th–75th percentile]**	**Median** **[25th–75th percentile]**	
Age (years)	60.50 [49.00–72.00]	61.00 [50.25–69.00]	0.67	61.00 [50.00–71.00]	59 [48.88–65.50]	0.57
DLCO (% predicted value)	75.00 [67.50–87.50]	80.00 [70.00–89.00]	0.39	79.00 [70.00–88.00]	72.00 [59.50–93.00]	0.42
FEV1 (% predicted value)	97.00 [90.00–104.50]	102.50 [93.50–1113.50]	**0.02**	102.00 [92.00–113.00]	95.00 [88.00–103.00]	**0.04**
FVC (% predicted value)	99.00 [89.25–106.75]	98.00 [90.00–111.00]	0.48	99.00 [90.00–109.00]	95.50 [90.50–101.00]	0.31
RSA total score	133.55 [113.00–145.00]	137.00 [120.00–148.75]	0.19	134.50 [120.00–147.00]	137.00 [102.75–149.25]	0.41
Length of hospital in-stay (days)	10 [5–18]	9 [5–16]	0.84	9 [5–16]	9 [4–16]	0.66

*BDI-II, Beck Depression Inventory-II; DLCO, diffusing capacity of the lung for carbon monoxide; FEV1, forced expiratory volume in the 1st second; FVC, forced vital capacity; RSA, Resilience Scale for Adults.*

*The P-values of significant findings are written in bold*.

When run for depressive symptoms at the psychiatric interview, the logistic regression highlighted statistically significant results, showing higher odds in females (OR 0.34; 95% CI 0.18–0.64; *p* = 0.0009), patients with COPD (OR 3.39; 95% CI 1.02–11.29; *p* = 0.04) and with persistent, poor tolerance to physical efforts at follow-up (OR 2.24; 95% CI 1.08–4.67; *p* = 0.03) (Table 6).

**Table 6:** Logistic regression model for depressive symptoms as assessed at the psychiatric interview and with the BDI-II.

	**Depressive symptoms at the clinical interview**			**BDI-II Total score (>13)**	
	**OR (95% CI)**	***p*-value**		**OR (95% CI)**	***p*-value**
Exercise intolerance/Poor tolerance to physical efforts	2.24 (1.08–4.67)	**0.03**	Exercise intolerance/Poor tolerance to physical efforts	3.39 (1.25–9.21)	**0.02**
History of anxiety and depression	2.66 (0.68–10.41)	0.16	History of anxiety and depression	11.10 (2.64–46.62)	**0.001**
Gender	0.34 (0.18–0.64)	** <0.001**	Gender	0.39 (0.15–1.03)	0.06
RSA Total Score	0.99 (0.98–1.01)	0.79	Obesity	4.03 (1.02–15.89)	**0.04**
Fev1	0.98 (0.97–1.01)	0.29	Fev1	0.97 (0.95–1.00)	0.09
Oxygen support	0.72 (0.49–1.08)	0.07	Diabetes	0.10 (0.01–1.04)	0.05
COPD	3.39 (1.02–11.29)	**0.046**			
Hypertension	0.65 (0.33–1.29)	0.22			

*BDI-II, Beck Depression Inventory; COPD, Chronic Obstructive Pulmonary Disease; FEV1, forced expiratory volume in the 1st second; RSA, Resilience Scale for Adults.*

*The P-values of significant findings are written in bold*.

### Depression According to BDI-II Scores

According to the BDI-II categorization described in the methods, compared to those with minimal depression according to the BDI-II score, a higher rate of patients in the mild to severe depression group was already treated by psychiatric services, had a previous history of anxiety/depression, and was sent for further psychiatric consultation. Furthermore, patients in the mild to severe group according to the BDI-II scores categorization more frequently showed anxiety at the clinical interview, as well as changes in sleep and appetite patterns since discharge from hospital (Table 4).

When dividing participants according to BDI-II scores (minimal vs. mild to severe depression) no difference was found in the socio-demographic and clinical continuous variables investigated (age, DLCO, FVC, IES score, RSA Total score), except for FEV1. Participants with mild to severe levels of depression according to the BDI-II had lower FEV1 values than those with minimal depression (*p* = 0.044, median 95 [88–103] vs. 102 [92–113]) (Table 5).

The logistic regression found associations between mild to severe BDI-II score, obesity (OR 4.03; 95% CI 1.02–15.89; *p* = 0.04), poor tolerance to physical efforts (OR 3.39; 95% CI 1.25–9.21; *p* = 0.02) and previous history of anxiety and/or depression (OR 11.10; 95% CI 2.6–46.62; *p* = 0.001) (Table 6).

## Discussion

In our sample, at the psychiatric assessment 32.9 and 29.5% of participants showed anxiety and depressive symptoms since hospital discharge, respectively. Changes in appetite and sleep patterns emerged for 15.6 and 31.2% of patients at follow-up. According to the self-administered questionnaires, 7.1% of participants had moderate-severe anxiety levels (BAI), while 10.5% had mild to severe depression (BDI-II). Thirty-two participants (13.5%) were already treated by psychiatric services and after the psychiatric interview and assessment, 26 (11%) participants were referred to further psychiatric consultation.

The prevalence rates we found were lower than those described by a recent systematic review and random-effects meta-analysis which reported the following pooled prevalence values: 45% for depression (95% CI: 37–54%, I2 = 96%), 47% for anxiety (95% CI: 37–57%, I2 = 97%), and 34% for sleeping disturbances (95% CI: 19–50%, I2 = 98%) (16). Nonetheless, this systematic review did not specify about follow-up timing, and the Authors underscored that according to the screening tools used, depression and anxiety prevalence estimates highly varied. Some recent reports about the mid-term psychiatric outcomes in COVID-19 patients described indeed a wide range of rates of depressive symptoms, from 4.3 to 37.3% (22, 23, 26, 27, 44, 45), as well as for anxiety ones, from 6.5 to 47.8% (22, 23, 26, 27, 44, 45). Nonetheless, the possibility to compare results from different studies may be hard: there are many inconsistencies concerning sample selection and follow-up approach; most of the available studies lack exhaustive information, also as far as psychiatric history and medications are concerned. Furthermore, in most studies they were just used self-administered questionnaires, while in others, as the one by Sykes, specific symptoms were assessed directly asking patients whether or not they were currently experiencing them (23). In our study, according to the psychiatrists' clinical assessment, 32.9% of the sample had anxiety symptoms while anxiety as measured by the BAI was moderate to severe in 7.1% of participants. Some considerations can be made about this finding: first, the BAI is not a diagnostic tool and certainly a self-administered questionnaire cannot equate a clinical diagnosis; second, questions in the BAI specifically refer to the previous 1 week, while in the context of the clinical interview a longer period of time was assessed to establish whether anxiety symptoms were indeed present or not; furthermore, it is possible that the factors influencing anxiety during clinical interview and anxiety as measured with the BAI might differ. For instance, as post-traumatic stress symptoms were higher in patients with moderate-severe BAI score, but not in those with anxiety at the clinical interview, we cannot exclude that they played a role. It has been suggested that post-traumatic stress might be better captured by new tools, developed *ad hoc* for the current pandemic, rather than by already existing ones (46), while similar considerations have not yet been raised for other psychological dimensions, symptoms or constructs.

As far as depression is concerned, the clinical interview highlighted depression in 29.5% of the sample, while 10.5% of participants showed mild to severe levels of depression according to the BDI-II. Findings emerging from the self-administered questionnaire (BDI-II) and the assessment of depressive symptoms at the clinical interview seemed more consistent than those regarding anxiety and were not correlated with post-traumatic symptoms (no correlation with the IES scale). In patients with clinically assessed depressive symptoms it was also more frequent a previous history of anxiety and/or depression and having already been treated by psychiatric services.

In any case, as could be expected, the outcome of the clinical interview was consistent with the choice to suggest patients further psychiatric consultations. Interestingly, the severity of acute COVID manifestations during previous inpatient treatment (e.g., ICU admission, type of oxygen support needed) showed no correlation with anxiety nor with depression, either clinically assessed or self-reported. Currently available studies about the mid-term psychiatric outcomes in COVID-19 patients have yielded mixed results concerning this issue. Huang found more problems in terms of anxiety and depression, in participants with greatest disease severity during hospital stay, compared to those who needed hospital admission but no oxygen supplementation (21). On the other hand, our results are consistent with other previous reports, which found no correlation between pneumonia severity, fatigue and psychological morbidities in COVID-19 patients (23, 27, 28, 44).

Interestingly, associations have been suggested between depression or anxiety and persistent breathlessness (27) or physical symptoms at follow-up (44). The existing literature thus seems to suggest that it might be the persistence of physical symptoms, especially respiratory ones, to play a stronger role in the maintenance of the mid-term mental health consequences of the COVID-19 infection, rather than the severity of the acute disease. Our findings concerning depression seem to support this hypothesis. Actually, in our sample patients with depressive symptoms at the clinical interview, as well as those with mild to severe levels of depression according to BDI-II scores, had lower FEV1 values than those without. Furthermore, we found that patients with depressive symptoms as assessed at the clinical interview, as well as those scoring higher on the BDI-II, had greater odds for persistent, poor tolerance for physical efforts.

Despite we failed to find any statistically significant results concerning resilience, it cannot be excluded that potentially protective factors, such as resilience, coping skills, post-traumatic growth, faith, and support from family and friends might play a role on the psychiatric outcome of the COVID-19 infection (28). To our knowledge only the study by Venturelli et al. (26) assessed resilience, and as in our sample, the great majority of respondents seemed to have enough resources to react. Nonetheless, the lack of baseline data and of other comparable studies does not currently allow the understanding of the possible role of a construct like the one of resilience in mediating persistent anxiety or depression symptoms and post-traumatic stress ones.

### Limitations

As already pointed out, a key limitation of our research was patients' recruitment and selection, with a large number of patients who denied participation after being contacted by phone; this might have generated a selection bias, and the actual proportion of subjects with functional impairment and/or psychological sequelae might have been lower if all had participated. Unfortunately, as for similar studies, no pre-COVID 19 baseline data were available. Nonetheless, differently by most of the available studies about the topic, we asked participants information about their previous psychiatric history and contact with mental health services, even though information about medication treatment (e.g., antidepressants) was not systematically gathered and hence was not available for the analysis. Furthermore, we did not limit our assessment to subjective, self-administered questionnaires, whose pathological scores cannot be equated to a clinical diagnosis, but we performed a face-to-face psychiatric interview. As far as the MINI interview is concerned, it should be underscored that some researchers have suggested that, compared to the SCID, the MINI interview could classify major depression more often (47).

We believe that our multi-disciplinary assessment and the specific, multi-faceted approach we adopted for the psychiatric follow-up of COVID patients, represent relevant strengths of our research project.

## Conclusion

Follow-up studies should aim to identify those patients who might benefit more from further referral and care. Briefly, according to our findings, the self-administered measure we used for anxiety assessment (BAI) did not seem to differentiate well those who were referred for further consultation and those who were not, while the one we adopted for depression assessment (BDI-II) performed better. As could be expected according to clinical experience, the psychiatric interview effectively differentiated further referrals. It is important an approach including both a clinical interview conducted by an experienced psychiatrist and the use of self-administered questionnaires. This double approach is more likely to capture the psychiatric symptoms of patients recovered from COVID-19, and eventually identify patients who might benefit for further referral. Nonetheless, the availability of resources should be considered, also in the context of the continuing COVID-19 related emergency. Interestingly, the severity of COVID acute manifestations showed no correlation with anxiety nor with depression, but the persistence of respiratory and physical symptoms, even after the acute phase, correlated with psychological and psychopathological symptoms. Further studies and longer follow-up duration will allow a better understanding of the complex relationship between residual physical symptoms, quality of life and psychological health. For this purpose, our research group is planning the 1-year follow-up involving the participants to the current study.

## Data Availability Statement

The raw data supporting the conclusions of this article will be made available by the authors, without undue reservation.

## Ethics Statement

The studies involving human participants were reviewed and approved by Comitato Etico Interaziendale Novara; IRB code CE 117/20. The patients/participants provided their written informed consent to participate in this study.

## No-More COVID Group

Gian Carlo Avanzi^1,2^, Marco Battaglia^1^, Emanuela Cadario^1^, Vincenzo Cantaluppi^1,2^, Luigi Mario Castello^1,2^, Elisa Clivati^2^, Martina Costanzo^1,2^, Alessandro Croce^1,2^, Carla De Benedittis^1,2^, Simona De Vecchi^2^, Francesco Gavelli^1,2^, Leonardo Grisafi^1^, Eyal Hayden^1^, Marco Invernizzi^1,2^, Paolo Marzullo^1,2^, Erica Matino^1,2^, Antonio Panero^1,2^, Elena Parachini^2^, Filippo Patrucco, ^1,2^ Giuseppe Patti^1,2^, Alice Pirovano,^1^ Cristina Rigamonti,^1,2^ Daniele Soddu ^1^ and Erika Zecca^1^

^1^Dipartimento di Medicina Traslazionale, Università del Piemonte Orientale UPO, Novara, Italy

^2^Azienda Ospedaliero Universitaria Maggiore della Carità, Novara, Italy.

## Author Contributions

MP, MB, AB, PB, PS, and PZ designed the study. CG, EG, GB, SB, VB, AF, MG, EG, AJ, LL, DM, MM, PP, and the NO MORE COVID GROUP recruited and assessed patients. MB performed the statistical analyses. CG and PZ drafted the manuscript. All authors revised the manuscript and contributed with relevant intellectual content.

## Conflict of Interest

The authors declare that the research was conducted in the absence of any commercial or financial relationships that could be construed as a potential conflict of interest.
